# Supplementary material for: Introduction to Genomic Analysis Workshop: A catalyst for engaging life-science researchers in high throughput analysis
Source: F1000Res. 2019 Jul 30;8:1221. [Version 1] doi: 10.12688/f1000research.19320.1 (PMC6774052; doi:10.12688/f1000research.19320.1)
Supplement: Peer review history

# Peer review: Reviewer response for version 1

**DOI:** 10.5256/f1000research.21179.r53168

**Published:** 2019-09-30

**Authors:** Michael Springer

**Affiliations:** 1 Department of Systems Biology, Harvard Medical School, Boston, MA, USA

There is a huge need for teaching resources in computational areas for life scientist. While there are a growing number of resources, it is far from saturated. The authors focus on an important sub-area, high performance computing with an eye towards genomic sequence analysis. This is a smart choice as sequencing data has become an increasingly common tool in the life science and students recognize the need to be able to hand such data.

 The authors deliver a course consisting of seven workshops, four core and three optional. All the resources are shared online. The results of the course and feedback is shared making the strength and areas of potential improvements clear.

 As someone who delivers similar content to graduate student, and who has been design a similar course, I found the online resources valuable and something that I could use to build on for my own class.

 As such, I believe the article is ready for indexing.

I confirm that I have read this submission and believe that I have an appropriate level of expertise to confirm that it is of an acceptable scientific standard.

# Peer review: Reviewer response for version 1

**DOI:** 10.5256/f1000research.21179.r53165

**Published:** 2019-09-24

**Authors:** Melanie I. Stefan, Richard Fitzpatrick

**Affiliations:** 1 Centre for Discovery Brain Sciences, University of Edinburgh, Edinburgh, UK

Biomedical research relies increasingly on the use of high-performance computing (HPC), but there is a gap in effective and timely HPC-training for practising biomedical researchers. This article describes the implementation and deployment of a modular HPC training programme. It is useful in two ways: First, the authors show that a thoughtfully designed and delivered course can achieve the goal of providing useful training on HPC that life scientists can apply to their own research. Second, by making all course materials available under a Creative Commons license, the authors have created an invaluable resource for instructors, HPC facility coordinators and self-directed learners worldwide. We also appreciated that the author share the full (de-identified) data from the participant survey.   

 We do feel that the article category "Opinion Article" does not do this work justice, since it describes a rigorously designed educational intervention and collection of resources, as well as presenting evaluation data. We leave it up to the editors to find a better suited category.

**Minor points:**

- The abstract should include information about the outcomes of the course.
- The very first sentence ("The era of genomics and DNA sequencing is being rapidly incorporated into life science research fields ...") should be re-phrased for clarity.
- Figure 1: A reader's interpretation of a pie chart depends on their ability to interpret areas on the pie chart as representing the underlying numbers. Making the pie chart three-dimensional and tilting it does not add any information. Worse yet, it distorts the information available.
- Consider using "gender" instead of sex, since this is very probably the information that has been collected from participants.
- In describing how the course works, we would have liked a bit more information about the teaching assistants, in particular what exactly their role was, how many there were, and how they were trained and prepared for the task.
- The response rate on the post-course survey was not bad for this type of survey, but there is still a sizable proportion of non-respondents. There should be a short discussion on how this may affect survey outcomes. There is some indication (e.g. Bacon *et al.*, Marketing Education Review, 2016 ^1^) that responders are typically at the more "extreme" ends of the student satisfaction spectrum, i.e. students who really liked or really disliked the course are most likely to respond. As a consequence, a higher response rate would lower the scores for classes with high scores and raise the scores for classes with low scores. But on the other hand, see Nowell *et al.* (International Review of Economics Education, 2014 ^2^) who tried to estimate non-response bias in online teaching evaluations and found it negligibly small. It may be useful for the reader to have at least an acknowledgement of the problem in the article.
- Reference 10 is to an erratum on a paper, not the paper itself. The erratum here is important, because it clarifies the first author's last name, but we feel that the original paper should also be referenced.
- The github.io site for the course is really nice, but we notice that the links to final recordings are sometimes broken and just take the user back to the main site.

We confirm that we have read this submission and believe that we have an appropriate level of expertise to confirm that it is of an acceptable scientific standard.

## References

[1] : Nonresponse Bias in Student Evaluations of Teaching. *Marketing Education Review*.2016;26(2) : 10.1080/10528008.2016.1166442 93-104 10.1080/10528008.2016.1166442

[2] : Non-response bias in student evaluations of teaching. *International Review of Economics Education*.2014;17: 10.1016/j.iree.2014.05.002 30-38 10.1016/j.iree.2014.05.002
